# Skeletal and Dentoalveolar Changes in Growing Patients Treated with Rapid Maxillary Expansion Measured in 3D Cone-Beam Computed Tomography

**DOI:** 10.3390/biomedicines11123305

**Published:** 2023-12-13

**Authors:** Peri Colino-Gallardo, Irene Del Fresno-Aguilar, Laura Castillo-Montaño, Carlos Colino-Paniagua, Hugo Baptista-Sánchez, Laura Criado-Pérez, Alfonso Alvarado-Lorenzo

**Affiliations:** 1 Department of Dentistry, Universidad Católica San Antonio de Murcia, 30107 Murcia, Spain; peri.colino@pgoucam.com (P.C.-G.); irene96aguilar@gmail.com (I.D.F.-A.); lauracastillomontano@gmail.com (L.C.-M.); carloscolinopaniagua@gmail.com (C.C.-P.); 2 Department of Oral Surgery, Universidad de Salamanca, 37007 Salamanca, Spain; baptistasanchez@gmail.com (H.B.-S.); kuki@usal.es (A.A.-L.)

**Keywords:** rapid maxillary expansion, CBCT, growing patients, molar angulation, dentoalveolar, orthodontics, skeletal change

## Abstract

The skeletal and dental effects of rapid maxillary expansion (RME) have been extensively studied, but high-quality research is still needed to determine the three-dimensional (3D) effects of RME. The aim of this study was to compare skeletal and dentoalveolar parameters through cone-beam computed tomography (CBCT) pre- (T1) and post-treatment (T2) with respect to RME. Twenty growing patients (mean age 10.7 years) were treated with a Hyrax-type expander. A 3D CBCT was performed at T1 and T2, measuring nasal width, maxillary width, palatal height, maxillary arch perimeter, angulation of the upper first molar, and intermolar width. The mean palatal suture opening was 2.85 ± 0.62 mm (*p* < 0.0001). Nasal width increased 1.28 ± 0.64 mm and maxillary width 2.79 ± 1.48 mm (*p* < 0.0001). In contrast, palatal height was reduced 0.65 ± 0.64 mm (*p* < 0.0001). Regarding arch perimeter, the radicular perimeter increased 2.89 ± 1.80 mm, while the coronal perimeter increased 3.42 ± 2.09 mm (*p* < 0.0001). Molar angulation increased 5.62 ± 3.20° for the right molar and 4.74 ± 2.22° for the left molar (*p* < 0.0001). Intermolar width increased 5.21 ± 1.55 mm (*p* < 0.0001). Treatment with Hyrax produced a significant opening in the mean palatal suture. Also, a significant increase in nasal width, maxillary width, arch perimeter, molar angulation, and intermolar width, and a decrease in palatal height, were observed.

## 1. Introduction

Maxillary transverse deficiency and crowding in children are problems commonly presented in orthodontic practices [1,2,3,4]. This palatal volume deficiency has been related to the volume of airways [5,6]. Patients with maxillary deficiency often have smaller nasal dimensions, tend to have greater resistance to airflow through the nose, and are often oral breathers, when compared to patients with a normal maxillary arch [7].

Rapid maxillary expansion (RME) is a method widely used to correct crossbites and maxillary deficiencies, especially as an early treatment in children [8,9,10,11]. In young adults, however, RME is limited by the maturation of the suture, so other types of appliances are commonly used to increase arch circumference, especially bone-borne appliances with micro-screws [12,13,14,15].

Clinical outcomes can often be different from what was anticipated. Frequently, when planning an RME, the midpalatal suture opening and the bone and dental tissue response have been expected based on the chronological age of the patient rather than the stage of suture maturation [16]. Patients show great variability in terms of the maturation of the midpalatal suture according to their chronological age [17,18]. This is why the use of CBCT (Cone-Beam Computed Tomography) prior to planning a rapid expansion of the maxilla is essential to determine the stage of the suture [19,20]. Hand–wrist X-rays [21] and the cervical vertebrae maturation stage method (CVM) [18] are also reliable methods commonly used to determine skeletal maturation.

Traditionally, studies about changes after RME have been based on occlusal radiographs and frontal cephalometry, as these are the means commonly used in diagnosis and during orthodontic treatment [22,23,24,25,26]. However, with the advent of CBCT, a more accurate and replicable assessment of anatomical structures in all three planes of space has been achieved [12,13,14,15,27,28,29,30,31,32,33,34,35]. On the other hand, there is an increasing interest in the evaluation of dental and skeletal changes as well as changes in the nasal cavity after RME with CBCT in growing patients [12,27,28,29,30,32,36,37,38,39,40,41,42,43,44]. However, most work in recent years has focused mainly on studying volumetric changes [29,30,36,37,40,43,45], but it is also important to analyze changes in linear dimensions that occur after RME [44].

The skeletal and dental effects of RME have been extensively studied [28,32,38,39,46,47,48,49,50], but the heterogeneity and quality of the available studies do not provide sufficient evidence; the correlation between dental and skeletal variables has not been sufficiently analyzed; and randomized controlled trials are needed to determine the three-dimensional effects of RME on the midpalatal suture [51]. Therefore, to date, information about the prediction of RME outcomes remains limited [52].

The aim of this study was to evaluate the skeletal and dentoalveolar changes of tooth-borne RME in growing patients in nasal and maxillary width, assessing changes at the upper first molars and establishing arch perimeter differences at both radicular and coronal levels.

## 2. Materials and Methods

### 2.1. Ethics Committee and Informed Consent

This research project was approved by the Bioethics Committee of the University of Salamanca (USAL_20/516). The study followed the guidelines established by the Declaration of Helsinki for research involving human subjects. All participants gave their informed consent before they were included in the study.

### 2.2. Sample Size and Participants

A prospective clinical study was conducted on a sample of 20 patients, 11 women and 9 men. The mean age of the sample was 10.7 years, with the oldest patient being 15.8 years old and the youngest 7.3 years old. The sample size was similar to those in previously published studies [9,27,28,41,42,43].

### 2.3. Study Design

**Figure 1:**
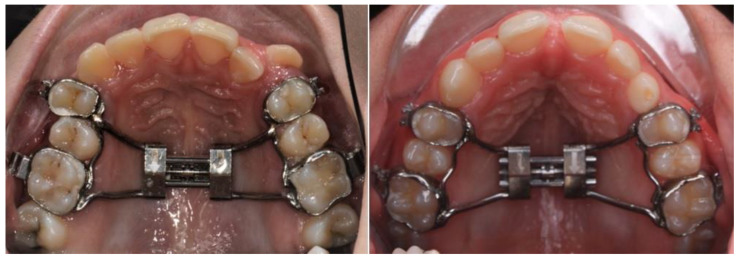
Hyrax tooth-borne expanders with 4 bands.

All patients were treated with a Hyrax expander, cemented in the upper first premolars and upper first molars (Figure 1). If the first premolars had not yet erupted, the Hyrax expander was cemented in the upper first deciduous molars and upper first permanent molars.

The activation protocol was the same in all patients: 2 × 1/4 turns (0.2 mm) per day until the desired sutural opening was obtained for each case, the average being about 15 days. A noticeable sutural opening was observed in all the patients in the sample, produced by the appearance of an interincisal diastema. The appearance of this sign is an expected result of treatment with RME, and it is accepted that there is a direct relationship between the opening degree of the interincisal diastema and the amount of orthopedic expansion [53].

Cone-beam computed tomographies (CBCT) were obtained before disjunction (T1) and after disjunction (T2). The parameters measured in each CBCT of the maxilla were: coronal arch perimeter (CP), root arch perimeter (RP), palatal height (PH), upper first molar angulation (MA), nasal base width (NBW), intermolar width (IMW), and maxillary width (JR–JL).

All participants met the following inclusion criteria: (1) Patients were included if they ranged in age from 7 to 15 years and were still growing according to the cervical vertebrae maturation method of Baccetti et al. (2005) [18]; (2) with skeletal maxillary compression; (3) with uni- or bilateral posterior crossbite; (4) with sufficient crown eruption to allow cementation of the RME; (5) with no family relationship to other patients participating in the study; (6) at growth stages CS3 or lower of the midpalatal suture according to Angelieri’s classification [54]; (7) absence of severe craniofacial syndromes or malformations; (8) absence of periodontal disease; (9) without agenesis; and (10) not having received previous orthopedic or orthodontic treatment.

### 2.4. Procedure

The study variables were measured by one operator on CBCT images taken with a GIANO 3D ADVANCED 13 × 16 (WhiteFox, Satelec, Merignac, France) with the following exposure parameters: 105.0 kV, 105.0 kV peak, 8.0 mA, and 7.20 s, with a field of view of 15 mm × 13 mm, and Anatomage Inc’s InVivo6 Dental software (Anatomage Europe, Milan, Italy) was used to perform the measurements. Each variable was measured before RME (T1) and after RME (T2).

**Figure 2:**
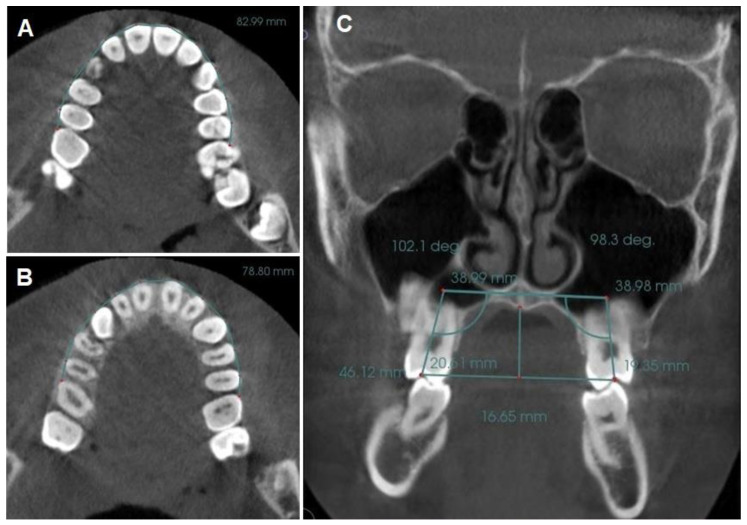
(**A**) Coronal perimeter (CP); (**B**) Root perimeter (RP); (**C**) Reference lines for palatal height (PH), 1st molar angulation (MA), and intermolar width (IMW).

The dentoalveolar variables analyzed were measured as follows:

1. Coronal perimeter (CP): the distance between the mesial of the right upper first molar and the mesial of the left upper first molar, passing through the vestibular side of all the teeth of the arch (Figure 2A).
2. Root perimeter (RP): the same procedure was used to measure the root perimeter but at the amelocemental junction level (ACJ) [8] (Figure 2B).
3. Angulation of the upper first molar (MA): the angle formed between a straight line drawn parallel to the hard palate plane (in sagittal view, utilizing the anterior nasal spine (ANS) and posterior nasal spine (PNS) as reference points) and a line passing through the center of the pulp chamber of both upper right and left first molars [12,55] (Figure 2C).
4. Intermolar width (IMW): the distance between the central fossa of the upper right and left first molars was measured (Figure 2C).

**Figure 3:**
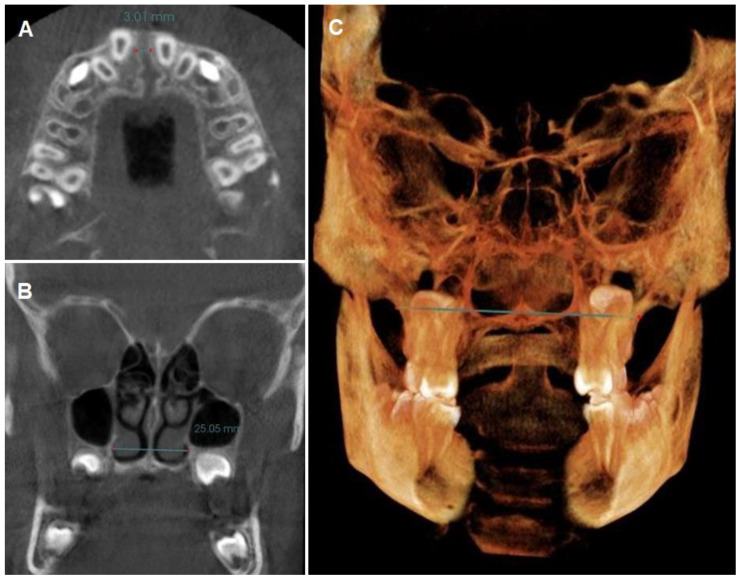
(**A**) Suture opening; (**B**) Nasal base width; (**C**) Maxillary width.

The skeletal variables analyzed were measured as follows:

1. Palatal height (PH): the distance from the midpalatal suture, tracing a perpendicular to the straight line formed from the central fossa of the right upper first molar to the central fossa of the left upper first molar [56] (Figure 2C).
2. Sutural opening (SO): a straight line was drawn from the right- to the left edges of the palatine suture at the incisal level, as this is where the greatest amount of disjunction occurs due to the fan-like opening pattern of the midpalatal suture after disjunction [57] (Figure 3A).
3. Nasal base width (NBW): the most posterior cut of the nasal cavity was taken, and a straight line was drawn from right to left from the base of the nasal cavity at its most inferior portion [58] (Figure 3B).
4. Maxillary width (JR–JL): the lowest point of intersection of the zygomatic bone with the maxillary tuberosity was taken from the patient’s right (JR) to the patient’s left (JL) [16] (Figure 3C).

T1 and T2 values were determined, and the difference between these two values was analyzed for each variable (Table 1).

### 2.5. Statistical Analysis

The data were analyzed using IBM SPSS Statistics (Version 29).

To determine a normal distribution of the variables, a Shapiro–Wilk test was performed, due to the small sample size. All variables fit a normal distribution. Only three differential values (Dif_RP, Dif_NBW, and Dif_MA) have a significance level slightly below 0.050. Once the normal distribution of the data was verified, a Student’s *t*-test for related samples was performed (Table 1). Levene’s test was conducted to compare the equality of variances for gender differences (Table 2). Two levels of significance were established: *p* < 0.05 as statistically significant and *p* < 0.01 as statistically highly significant.

## 3. Results

### 3.1. Differences between Measurements before RME (T1) and after RME (T2)

The mean age before treatment was 10.7 years and 11 years after treatment. On the other hand, to study the changes in measurements over time, Table 1 shows the Student’s *t*-test analyses for related samples. A statistically significant change was observed in all variables (Table 1). Likewise, a statistically significant difference was observed between males and females in left molar angulation, which was greater in females. However, the rest of the variables showed no significant differences in terms of gender (Table 2).

### 3.2. Correlation between Variables

To study the relationship between the variables, Table 3 shows the Pearson correlation matrix (or Spearman in the case of variables that do not comply with the normality assumption).

Two main results are observed:

Changes in CP and RP present the highest correlation between variables (r = 0.626; *p* < 0.01). In turn, changes in RP are related to JR–JL (r = 0.446; *p* < 0.05) and SO (r = 0.726; *p* < 0.01). And changes in CP are related to SO (r = 0.726; *p* < 0.01). In summary, these measures are positively related to each other, so an intervention on one of them implies an intervention on the other ones.

IMW and MA are significantly correlated (r = 0.454; *p* < 0.05, and r = 0.488; *p* < 0.05), so given an increasing IMW after intervention, MA has increased.

All transversal dental and skeletal variables showed a significant increase, indicating that maxillary expansion was satisfactorily achieved.

Regarding the arch perimeter in T1 and T2, both at the radicular and coronal level, both variables increased, which was also statistically significant (*p* < 0.0001 in both cases), being also significantly correlated (r = 0.626; *p* < 0.01). On average, RP increased by 2.89 ± 1.80 mm, while CP increased by 3.42 ± 2.09 mm.

Changes in JR–JL were related to RP but not to CP. Despite not finding a significant correlation between JR–JL and CP, upon analyzing the regression model, it is estimated that, for each millimeter gained in maxillary width (JR–JL), the CP increased by 0.45mm.

In relation to the upper first molars, the MA was significantly increased (*p* < 0.0001), as was the IMW (*p* < 0.0001). The MA increased on average 5.62 ± 3.20° for the right molar and 4.74 ± 2.22° for the left molar, in relation to the root–lingual torque, while the IMW increased by 5.21 ± 1.55mm. Both variables have a significant relationship (r = 0.454; *p* < 0.05, and r = 0.488; *p* < 0.05), where, according to Cohen’s statistic, the effect size was larger for IMW (3.36) than for MA (1.75 right molar/2.14 left molar).

Both NBW and maxillary width (JR–JL) were statistically significantly increased (*p* < 0.0001 in both cases). NBW increased, on average, 1.28 ± 0.64mm, while JR–JL increased an average of 2.79 ± 1.48mm. Both measures are linearly independent so that an increase in one of the parameters does not imply an increase in the other, and vice versa. Likewise, a statistically significant reduction in PH was observed after expansion, averaging 0.65 ± 0.64mm.

The mean palatal suture also increased significantly (*p* < 0.0001). The mean palatal suture opening was 2.85 ± 0.62mm. This variable showed a significant relationship with the increase in CP (r = 0.558; *p* < 0.05) and with the increase in RP (r = 0.726; *p* < 0.01).

Although there was no significant relationship between any of the variables and age, there was a tendency (a negative correlation) for the change to be greater the younger the age of the patients. This trend was observed in all the variables except for IMW and the left molar angulation (MA).

## 4. Discussion

The effects of RME have been extensively studied [16,32,59,60,61,62,63]. The ratio between the increase in transverse dimension and the dental changes resulting (arch perimeter, intermolar width, etc.) are useful to help plan orthodontic treatments, as they are often associated with the decision of whether or not to perform extractions. It is therefore of interest to the clinician to know what dental changes occur with RME and how much space can be gained in the dental arch with RME [8,9,59,64,65]. The size of the midpalatal suture opening will depend on the occlusal needs of each patient. In our study, a mean midpalatal suture opening (SO) of 2.85 ± 0.62mm was observed, which was statistically significant, and we found that this SO was related to an increase in arch perimeter (CP and RP).

- DENTOALVEOLAR CHANGES:

The measurement of molar angulations and intermolar width through CBCT is an innovative way to analyze orthodontic cases from a more accurate point of view compared to model analysis. RME generates changes in intermolar width (IMW), which in our study was increased by 5.21 ± 1.55 mm, values that coincide with those observed in most studies, which determine an increase of between 5.03mm and 6.7mm in intermolar width [8,9,12,13,60,61,65,66,67]. On the other hand, these data vary greatly from those obtained by Abdalla et al. (2019) [62], also due to measurement differences between studies, although the results are similar in terms of perimeter increase after disjunction. Other authors [16,59,62] also find an increase in intermolar width after RME, although slightly lower than that obtained in our study, observing an increase of between 4mm and 4.87 mm, and lower values are found by El and Palomo (2014) [30] and Canuto et al. (2010) [67], with 2.9 mm in both studies, although still significant. On the other hand, Halicioglu et al. (2010) [68] observed the highest values with an increase in intermolar width of 8.5mm. These discrepancies in measurements between studies confirm the lack of standardization of measurements.

Molar inclination has been described as a common side effect of RME [38,60,69,70,71,72,73,74,75]. In our study, molar angulation (MA) increased on average 5.62° for the right molar and 4.74° for the left molar. Similar values were found by other authors [12,59], with an increase of 4.7–4.8°, and were slightly lower than those of Adkins et al. (1990) [8], with a change of 7.3°, although they also obtained a wide standard deviation, ± 5.8°, compared to 3.20/2.22° in our study. Other authors [60] obtained higher values, with 21° of molar angulation; however, they do not take into account the angulation of each molar independently, so the results are not comparable. In addition, we found that left molar angulation was higher in females (5.93 ± 1.68°) than in males (3.28 ± 1.95°).

In our study, an increase in IMW is related with MA but not with maxillary width (JR–JL), which could mean that RME produces mainly dental changes. Adkins et al. (1990) [8] observed that, in patients with bilateral crossbite, a greater molar inclination occurs after RME than in patients without crossbite because, at a certain time of the treatment, the palatal slope of the palatal cusps of the maxillary teeth occludes with the vestibular slope of the lingual cusps of the mandibular teeth, generating an occlusal force that favors the buccal tip of the maxillary teeth.

RME also produces an increase in arch perimeter, both at the radicular and coronal levels. The root perimeter (RP) increased by 2.89 ± 1.80mm, while the coronal perimeter (CP) increased by 3.42 ± 2.09mm, where we obtained similar values to those observed in other studies [8,9,62]. These parameters significantly correlated with each other and also correlated directly with the opening of the midpalatal suture. Other authors found higher values, with an increase of between 4.1mm and 5.05mm of CP [66,76]. McNamara et al. (2003) [59] found even higher values, with a mean value of 6.3mm, and Aparecida et al. (2006) [65] and Canuto et al. (2010) [67] found the lowest values, with a mean of 2.41mm and 2.69mm of PC increase, respectively.

Knowing the proportion in which the maxillary width or intermolar width increases with respect to the increase in CP, one could estimate the amount of spatial increase in arch perimeter that we will obtain according to the amount of maxillary expansion performed. Thus, for an average of 4.4mm of molar expansion, McNamara et al. (2003) [59] found a gain of approximately 6mm in arch perimeter (CP). In our study, despite not finding a significant correlation between the variables, by analyzing the regression model, it is estimated that, for each millimeter gained in maxillary width, the arch perimeter increases by 0.45mm. Adkins et al. (1990) [8] observed that the increase in arch perimeter can be predicted as 0.7 times the amount of expansion performed; however, Berlocher et al. (1980) [64] observed an increase of 1/1. These results can be used as a guideline for estimating the increase in the perimeter after RME.

- SKELETAL CHANGES:

Regarding the increase in maxillary width (JR–JL), Pereira et al. (2017) [60] obtained an increase of 1.76 mm, slightly lower data figures than our results, where we observed an increase of 2.79 ± 1.48 mm. The most similar data are found by Abdalla et al. (2019) [62] and Sayar and Kılınç (2019) [16] with a 2.29–2.91 mm increase in maxillary width. In contrast, El and Palomo (2014) [30] observe higher values of 3.5mm of increase in maxillary width.

In previous studies [30,33,61], it has been observed that the size of the nasal structures is affected by the expansion of the maxilla, and the nasal base width (NBW) increases between 1.7mm and 2.39mm; in our study, we found similar values to those studies, with an increase of 1.28 ± 0.64mm on average. The authors agree that the changes observed in the studies are small, and the standard deviations are wide [8].

Kinzinger et al. (2022) [63] argue that the interaction of the different centers of rotation of the palate during RME is the reason for the changes in palatal height and palatal shape after RME. Especially the centers of rotation in the frontal plane, near the frontomaxillary sutures, originate the rotation of the hard palate, which pivots laterally, generating an increase in palatal height. However, in the present study, we observed a significant reduction in palatal height (PH) after an expansion of 0.65 ± 0.65 mm on average. However, the way the values were measured differed from one study to another.

To interpret all these data, it is necessary to take into account the natural growth of the maxilla without RME treatment. It is difficult to quantify the amount of skeletal expansion that is exclusively due to RME expansion because it is usually performed in preadolescents, so the long-term effects are a combination of the treatment and the patient’s natural growth [77]. What we knew until recently about maxillary growth was based on older studies using implants, frontal cephalometry, and model analysis, with many limitations [77,78,79,80,81]. However, Seubert et al. (2021) [77] confirm that the results obtained in these studies are comparable to those obtained with the technological means currently available (CBCT). Thus, the classic studies by Björk [78,79] estimated a transverse growth of 0.42 ± 0.12 mm per year; Korn and Baumrind (1990) [80] observed a similar growth of 0.51 ± 0.16 mm per year; and recent studies with CBCT [77] confirm an annual transverse growth of 0.50 ± 0.31 mm. Regarding nasal width, Seubert et al. (2021) [77] observed an increase of 0.3mm per year. All this indicates that a small part of the growth observed in any growing sample is due to the normal growth of the patient.

When comparing these values with those observed in studies using different expansion appliances, studies using mini-screw-assisted rapid palatal expansion (MARPE) or surgically assisted rapid palatal expansion (SARPE) found similar values to those found in our study with RME, with 5.34–5.8 mm of IMW increase [12,82], while other studies found lower values of 3.70–4.91 mm [13,14]. The higher values of IMW increase were observed by Altug et al. (2006) [83] with 7.81 for both RME and SARPE groups, and other studies showed lower increases of 0.98–2.2mm using slow maxillary expansion (SME) appliances [33,35]. On the other hand, it was observed that MA was lower in studies using MARPE and SARPE [12,14,15,82], except for Altug et al. (2006) [83] who found higher MA in the SARPE group than in the RME group, and the MA had higher values in the SME group when comparing it to the RME group [33], although their MA values in the RME group were lower than in our study. Regarding the skeletal parameters, studies found the higher increases in the NBW with MARPE and SARPE and the lowest increases with SME [12,14,15,33]. Similar values to those observed in our study for maxillary width (JR–JL) and SO with RME were observed in studies with MARPE and SARPE [12,15,83]. However, we have to keep in mind the lack of standardization of measurements when interpreting these differences or similarities between studies. Some studies also evaluate alveolar bone changes after expansion with different outcomes, which would be interesting to include in future investigations [12,14,15,34,35].

- LIMITATIONS OF THIS STUDY:

This study has a number of limitations. Although the measurements have shown significant differences, the sample size is small. Also, a comparison with patients without growth could be carried out, as the lack of a control group makes it difficult to know whether the observed changes are due to a patient’s own growth or to the effect of RME. On the other hand, the lack of standardization of CBCT measurements makes it difficult to compare between similar studies, which coincides with what has been observed in other analyses [51]. The method error has not been assessed. The measurements studied can be reproduced but could differ according to the operator, due to the fact that the establishment of reference points on the CBCT is not automatic, and the operator must choose where to place them. For this reason, there could be an increase in inter- and intra-operator error when the same cases are studied.

## 5. Conclusions

According to the results observed in the present study, we can conclude that tooth-borne RME produces an increase in nasal width and maxillary width and also in the radiculo–lingual torque of the upper molars and in the intermolar width. Tooth-borne RME also produces an increase in arch perimeter, both at the coronal and the radicular level. Although there is no significant relationship with the increase in the coronal perimeter, the increase in maxillary width shows a tendency to increase in a proportion of 1/0.45 in relation to the increase in coronal perimeter (JR–JL/CP). This may serve as an estimation of the space that can be gained after RME.

## Figures and Tables

**Table 1:** Comparison of dentoalveolar and skeletal variables studied at T1 and T2.

	T1 (Mean)	T2 (Mean)	Difference T2–T1	SD	IC95%	T	*p*-Value	D
Dentoalveolar variables								
CP (mean) (mm)	82.97	86.4	3.42	2.09	[2.45–4.40]	7.34	<0.0001 **	1.64
RP (mean) (mm)	80.48	83.33	2.89	1.80	[2.04–3.73]	7.18	<0.0001 **	1.61
MA (mean) (degree)	99.41/ 99.58	105.03/ 104.32	5.62/ 4.74	3.20/ 2.22	[4.12–7.11]/ [3.70–5.78]	7.84/9.56	<0.0001/<0.0001 **	1.75/ 2.14
IMW (mean) (mm)	44.52	49.73	5.21	1.55	[4.48–5.93]	15.03	<0.0001 **	3.36
Skeletal variables								
SO (mean) (mm)	0	2.85	2.85	0.62	[2.57–3.14]	20.84	<0.0001 **	4.66
NBW (mean) (mm)	25.24	26.52	1.28	0.64	[0.98–1.57]	8.99	<0.0001 **	2.01
PH (mean) (mm)	15.79	15.13	−0.65	0.64	[−0.95–−0.35]	−4.56	<0.0001 **	1.02
JR–JL (mean) (mm)	59.68	62.47	2.79	1.48	[2.10–3.48]	8.43	<0.0001 **	1.89

SD: standard deviation. ** Statistically significant results (*p* < 0.01). CP: Coronal perimeter. RP: Root perimeter. MA: Angulation of the 1st molar (right molar/left molar). IMW: Intermolar width. SO: Suture opening. NBW: Nasal base width. PH: Palatal height. JR–JL: Maxillary width JR–JL.

**Table 2:** Gender comparison of changes at T2 in dentoalveolar and skeletal variables.

	Male Mean (SD)	Female Mean (SD)	*p*-Value
DIF_CP	3.51 (1.95)	3.34 (2.28)	0.863
DIF_RP	2.35 (1.59)	3.32 (1.90)	0.238
DIF_NBW	1.06 (0.71)	1.45 (0.53)	0.178
DIF_IMW	5.20 (1.64)	5.21 (1.54)	0.989
DIF_PH	−0.65 (0.58)	−0.65 (0.70)	0.999
DIF_MA Right molar	5.21 (3.62)	5.94 (2.94)	0.623
DIF_MA Left molar	3.28 (1.95)	5.93 (1.68)	0.004 **
DIF_JR–JL	2.68 (1.22)	2.87 (1.71)	0.789
SO_T2	2.92 (0.53)	2.79 (0.68)	0.634

SD: standard deviation. ** Statistically significant results (*p* < 0.01). CP: Coronal perimeter. RP: Root perimeter. NBW: Nasal base width. IMW: Intermolar width. PH: Palatal height. MA: Angulation of the 1st molar (right molar/left molar). JR–JL: Maxillary width JR–JL. SO: Suture opening.

**Table 3:** Correlation matrix between the differential variables under study.

	AGE_T2	Dif_CP	Dif_RP	Dif_ NBW	Dif_ IMW	Dif_PH	Dif_ MA	Dif_ JR–JL	SO_T2
AGE_T2	1								
Dif_CP	−0.118	1							
Dif_RP	−0.189	0.626 **	1						
Dif_ NBW	−0.226	*0.189*	*−0.058*	1					
Dif_ IMW	0.113	0.205	*0.135*	*0.217*	1				
Dif_PH	−0.099	−0.177	*−0.242*	*−0.357*	−0.434	1			
Dif_MA	−0.129/ 0.067	*−0.063/* *0.066*	*−0.027/* *0.200*	*−0.019/* *0.319*	0.454 */ 0.488 *	*−0.234/* *−0.257*	1		
Dif_ JR–JL	−0.029	0.317	0.446 *	*−0.037*	0.346	−0.400	0.091/ 0.133	1	
SO_T2	−0.165	0.558 *	0.726 **	0.099	−0.080	−0.126	−0.029/ −0.057	0.091	1

SD: standard deviation. * Statistically significant results (*p* < 0.05). ** Statistically significant results (*p* < 0.01). Spearman correlations are marked in italics. CP: Coronal perimeter. RP: Root perimeter. NBW: Nasal base width. IMW: Intermolar width. PH: Palatal height. MA: Angulation of the 1st molar (right molar/left molar). JR–JL: Maxillary width JR–JL. SO: Midpalatal suture opening.

## Data Availability

Data are only available on request, due to restrictions, e.g., privacy or ethical.

## References

[1] Brunelle J.A., Bhat M., Lipton J.A. (1996). Prevalence and distribution of selected occlusal characteristics in the US population, 1988–1991. J. Dent. Res..

[2] Ciuffolo F., Manzoli L., D’Attilio M., Tecco S., Muratore F., Festa F., Romano F. (2005). Prevalence and distribution by gender of occlusal characteristics in a sample of Italian secondary school students: A cross-sectional study. Eur. J. Orthod.

[3] Gabris K., Marton S., Madlena M. (2006). Prevalence of malocclusions in Hungarian adolescents. Eur. J. Orthod..

[4] Da Silva Filho O.G., Santamaria M., Capelozza Filho L. (2007). Epidemiology of posterior crossbite in the primary dentition. J. Clin. Pediatr. Dent..

[5] Lowe A.A. (1990). The tongue and airway. Otolaryngol. Clin. North Am..

[6] Pae E.K., Lowe A.A., Sasaki K., Price C., Tsuchiya M. (1994). A cephalometric and electromyographic study of upper airway structures in the upright and supine positions. Am. J. Orthod. Dentofac. Orthop..

[7] Subtelny J.D. (1980). Oral respiration: Facial maldevelopment and corrective dentofacial orthopedics. Angle Orthod..

[8] Adkins M.D., Nanda R.S., Currier G.F. (1990). Arch perimeter changes on rapid palatal expansion. Am. J. Orthod. Dentofac. Orthop..

[9] D’Souza I.M., Kumar H.C., Shetty K.S. (2015). Dental arch changes associated with rapid maxillary expansion: A retrospective model analysis study. Contemp Clin Dent..

[10] Khosravi M., Ugolini A., Miresmaeili A., Mirzaei H., Shahidi-Zandi V., Soheilifar S., Karami M., Mahmoudzadeh M. (2019). Tooth-borne versus bone-borne rapid maxillary expansion for transverse maxillary deficiency: A systematic review. Int. Orthod..

[11] An J.S., Seo B.Y., Ahn S.J. (2021). Comparison of dentoskeletal and soft tissue changes between tooth-borne and tooth-bone-borne hybrid nonsurgical rapid maxillary expansions in adults: A retrospective observational study. BMC Oral Health..

[12] Bazargani F., Lund H., Magnuson A., Ludwig B. (2021). Skeletal and dentoalveolar effects using tooth-borne and tooth-bone-borne RME appliances: A randomized controlled trial with 1-year follow-up. Eur. J. Orthod..

[13] Lagravère M.O., Ling C.P., Woo J., Harzer W., Major P.W., Carey J.P. (2020). Transverse, vertical, and anterior-posterior changes between tooth-anchored versus Dresden bone-anchored rapid maxillary expansion 6 months post-expansion: A CBCT randomized controlled clinical trial. Int. Orthod..

[14] Moon H.W., Kim M.J., Ahn H.W., Kim S.J., Kim S.H., Chung K.R., Nelson G. (2020). Molar inclination and surrounding alveolar bone change relative to the design of bone-borne maxillary expanders: A CBCT study. Angle Orthod..

[15] Solano P., Aceytuno P., Solano E., Solano B. (2022). Skeletal, dentoalveolar and dental changes after “mini-screw assisted rapid palatal expansion” evaluated with cone beam computed tomography. J. Clin. Med..

[16] Sayar G., Kılınç D.D. (2019). Rapid maxillary expansion outcomes according to midpalatal suture maturation levels. Prog. Orthod..

[17] Fishman L.S. (1982). Radiographic evaluation of skeletal maturation. A clinically oriented study based on hand-wrist films. Angle Orthod..

[18] Baccetti T., Franchi L., McNamara J.A. (2005). The cervical vertebral maturation (CVM) method for the assessment of optimal treatment timing in dentofacial orthopedics. Semin. Orthod..

[19] Korbmacher H., Schilling A., Püschel K., Amling M., Kahl-Nieke B. (2007). Age-dependent three-dimensional microcomputed tomography analysis of the human midpalatal suture. J. Orofac. Orthop..

[20] Angelieri F., Cevidanes L.H., Franchi L., Gonçalves J.R., Benavides E., McNamara J.A. (2013). Midpalatal suture maturation: Classification method for individual assessment before rapid maxillary expansion. Am. J. Orthod. Dentofac. Orthop..

[21] Flores–Mir C., Nebbe B., Major P.W. (2004). Use of skeletal maturation based on hand-wrist radiographic analysis as a predictor of facial growth: A systematic review. Angle Orthod..

[22] Haas A.J. (1961). Rapid expansion of the maxillary dental arch and nasal cavity by opening the midpalatal suture. Angle Orthod..

[23] Wertz R.A. (1968). Changes in nasal airflow incident to rapid maxillary expansion. Angle Orthod..

[24] Warren D.W., Turvey T.A., Hairfield W.M. (1987). The nasal airway following maxillary expansion. Am. J. Orthod. Dentofac. Orthop..

[25] Cross D.L., McDonald J.P. (2000). Effect of rapid maxillary expansion on skeletal, dental, and nasal structures a postero-anterior cephalometric study. Eur. J. Orthod..

[26] Melo M.F., Melo S.L., Zanet T.G., Fenyo-Pereira M. (2013). Digital radiographic evaluation of the midpalatal suture in patients submitted to rapid maxillary expansion. Indian J. Dent. Res..

[27] Haralambidis A., Ari-Demirkaya A., Acar A., Küçükkeleş N., Ateş M., Ozkaya S. (2009). Morphologic changes of the nasal cavity induced by rapid maxillary expansion: A study on 3-dimensional computed tomography models. Am. J. Orthod. Dentofac. Orthop..

[28] Christie K.F., Boucher N., Chung C.H. (2010). Effects of bonded rapid palatal expansion on the transverse dimensions of the maxilla a cone-beam computed tomography study. Am. J. Orthod. Dentofac. Orthop..

[29] Caprioglio A., Meneghel M., Fastuca R., Zecca P.A., Nucera R., Nosetti L. (2014). Rapid maxillary expansion in growing patients correspondence between 3-dimensional airway changes and polysomnography. Int. J. Pediatr. Otorhinolaryngol..

[30] El H., Palomo J.M. (2014). Three-dimensional evaluation of upper airway following rapid maxillary expansion a CBCT study. Angle Orthod..

[31] Woller J.L., Kim K.B., Behrents R.G., Buschang P.H. (2014). An assessment of the maxilla after rapid maxillary expansion using cone beam computed tomography in growing children. Dental Press J. Orthod..

[32] Lin L., Ahn H.W., Kim S.J., Moon S.C., Kim S.H., Nelson G. (2015). Tooth-borne vs bone-borne rapid maxillary expanders in late adolescence. Angle Orthod..

[33] Serafin M., Fastuca R., Caprioglio A. (2022). CBCT analysis of dento-skeletal changes after rapid versus slow maxillary expansion on deciduous teeth: A randomized clinical trial. J. Clin. Med..

[34] Baysal A., Uysal T., Veli I., Ozer T., Karadede I., Hekimoglu S. (2013). Evaluation of alveolar bone loss following rapid maxillary expansion using cone-beam computed tomography. Korean J. Orthod..

[35] Bahammam M., El-Bialy T. (2023). Comparison of alveolar bone thickness and height after slow expansion using quad-helix or clear aligners. Saudi Dent. J..

[36] Palaisa J., Ngan P., Martin C., Razmus T. (2007). Use of conventional tomography to evaluate changes in the nasal cavity with rapid palatal expansion. Am. J. Orthod. Dentofac. Orthop..

[37] Felippe N.L.O., Silveira A.C., Viana G., Kusnoto B., Smith B., Evans C.A. (2008). Relationship between rapid maxillary expansion and nasal cavity size and airway resistance short- and long-term effects. Am. J. Orthod. Dentofacial Orthop..

[38] Garrett B.J., Caruso J.M., Rungcharassaeng K., Farrage J.R., Kim J.S., Taylor G.D. (2008). Skeletal effects to the maxilla after rapid maxillary expansion assessed with cone-beam computed tomography. Am. J. Orthod. Dentofac. Orthop..

[39] Lione R., Ballanti F., Franchi L., Cozza P. (2008). Treatment and posttreatment skeletal effects of rapid maxillary expansion studied with low-dose computed tomography in growing subjects. Am. J. Orthod. Dentofac. Orthop..

[40] Zhao Y., Nguyen M., Gohl E., Mah J.K., Sameshima G., Enciso R. (2010). Oropharyngeal airway changes after rapid palatal expansion evaluated with cone-beam computed tomography. Am. J. Orthod. Dentofac. Orthop..

[41] Pangrazio-Kulbersh V., Wine P., Haughey M., Pajtas B., Kaczynski R. (2012). Cone beam computed tomography evaluation of changes in the naso-maxillary complex associated with two types of maxillary expanders. Angle Orthod..

[42] Chang Y., Koenig L.J., Pruszynski J.E., Bradley T.G., Bosio J.A., Liu D. (2013). Dimensional changes of upper airway after rapid maxillary expansion: A prospective cone-beam computed tomography study. Am. J. Orthod. Dentofac. Orthop..

[43] Bouserhal J., Bassil-Nassif N., Tauk A., Will L., Limme M. (2014). Three-dimensional changes of the naso-maxillary complex following rapid maxillary expansion. Angle Orthod..

[44] Caldas L.D., Takeshita W.M., Machado A.W., Bittencourt M.A.V. (2020). Effect of rapid maxillary expansion on nasal cavity assessed with cone-beam computed tomography. Dental Press J. Orthod..

[45] Buck L.M., Dalci O., Darendeliler M.A., Papageorgiou S.N., Papadopoulou A.K. (2017). Volumetric upper airway changes after rapid maxillary expansion a systematic review and meta-analysis. Eur. J. Orthod..

[46] Haas A.J. (1965). The treatment of maxillary deficiency by opening the mid-palatal suture. Angle Orthod..

[47] Babacan H., Sokucu O., Doruk C., Ay S. (2006). Rapid maxillary expansion and surgically assisted rapid maxillary expansion effects on nasal volume. Angle Orthod..

[48] Doruk C., Sökücü O., Biçakçi A.A., Yilmaz U., Taş F. (2007). Comparison of nasal volume changes during rapid maxillary expansion using acoustic rhinometry and computed tomography. Eur. J. Orthod..

[49] Conroy-Piskai C., Galang-Boquiren M.T., Obrez A., Viana M.G., Oppermann N., Sanchez F., Edgren B., Kusnoto B. (2016). Assessment of vertical changes during maxillary expansion using quad helix or bonded rapid maxillary expander. Angle Orthod..

[50] Canan S., Şenışıkm N.E. (2017). Comparison of the treatment effects of different rapid maxillary expansion devices on the maxilla and the mandible. Part 1: Evaluation of dentoalveolar changes. Am. J. Orthod. Dentofac. Orthop..

[51] Liu S., Xu T., Zou W. (2015). Effects of rapid maxillary expansion on the midpalatal suture a systematic review. Eur. J. Orthod..

[52] Angelieri F., Franchi L., Cevidanes L.H., Bueno-Silva B., McNamara J.A. (2016). Prediction of rapid maxillary expansion by assessing the maturation of the midpalatal suture on cone beam CT. Dental Press J. Orthod..

[53] Ribeiro G.L.U., Locks A., Pereira J., Brunetto M. (2010). Analysis of rapid maxillary expansion using cone-beam computed tomography. Dental Press J. Orthod..

[54] Angelieri F., Franchi L., Cevidanes L.H., McNamara J.A. (2015). Diagnostic performance of skeletal maturity for the assessment of midpalatal suture maturation. Am. J. Orthod. Dentofacial Orthop..

[55] Yan-Cheng L., Kwok-Hing H., Chih-Wei W., Kai-Long W., Shun Chu H., Heng-Ming C. (2022). Skeletal and dental changes after microimplant-assisted rapid palatal expansion (MARPE)—A Cephalometric and Cone-Beam Computed Tomography (CBCT) study. Clin. Investig. Orthod..

[56] El Nahass H., Naiem S.N. (2016). Palatal bone dimensions on cone beam computed tomography. Implications for the palate as autogenous donor site: An observational study. Int. J. Oral Maxillofac. Surg..

[57] Bell R.A. (1982). A review of maxillary expansion in relation to rate of expansion and patient’s age. Am. J. Orthod..

[58] Moreira A.M., de Menezes L.M., Roithmann R., Rizzatto S.M.D., Weissheimer A., Yen S.L., Enciso R., de Lima E.M.S., Azeredo F. (2017). Immediate effects of rapid maxillary expansion on the nasal cavity using Haas-type and Hyrax-type expanders in CBCT. Med. Clin. Arch..

[59] McNamara J.A., Baccetti T., Franchi L., Herberger T.A. (2003). Rapid maxillary expansion followed by fixed appliances: A long-term evaluation of changes in arch dimensions. Angle Orthod..

[60] Pereira J.D.S., Jacob H.B., Locks A., Brunetto M., Ribeiro G.L.U. (2017). Evaluation of the rapid and slow maxillary expansion using cone-beam computed tomography: A randomized clinical trial. Dental Press J. Orthod..

[61] Fastuca R., Lorusso P., Lagravère M.O., Michelotti A., Portelli M., Zecca P.A., Antò V.D., Militi A., Nucera R., Caprioglio A. (2017). Digital evaluation of nasal changes induced by rapid maxillary expansion with different anchorage and appliance design. BMC Oral Health.

[62] Abdalla Y., Brown L., Sonnesen L. (2019). Effects of rapid maxillary expansion on upper airway volume: A three-dimensional cone-beam computed tomography study. Angle Orthod..

[63] Kinzinger G.S.M., Hourfar J., Buschhoff C., Heller F., Korbmacher-Steiner H.M., Lisson J.A. (2022). Age-dependent interactions of maxillary sutures during RME and their effects on palatal morphology. J. Orofac. Orthop..

[64] Berlocher W.C., Mueller B.H., Tinaoff N. (1980). The effect of maxillary palatal expansion on the primary dental arch circumference. Pediatr. Dent..

[65] Aparecida C.C., Abrão J., Reis S.A., de Fantini S.M. (2006). Correlation between transverse expansion and increase in the upper arch perimeter after rapid maxillary expansion. Braz. Oral. Res..

[66] Moussa R., O’Reilly M.T., Close J.M. (1995). Long-term stability of rapid palatal expander treatment and edgewise mechanotherapy. Am. J. Orthod. Dentofac. Orthop..

[67] Canuto L.F., de Freitas M.R., Janson G., de Freitas K.M., Martins P.P. (2010). Influence of rapid palatal expansion on maxillary incisor alignment stability. Am. J. Orthod. Dentofac. Orthop..

[68] Halicioglu K., Kiliç N., Yavuz I., Aktan B. (2010). Effects of rapid maxillary expansion with a memory palatal split screw on the morphology of the maxillary dental arch and nasal airway resistance. Eur. J. Orthod..

[69] Haas A.J. (1970). Palatal expansion: Just the beginning of dentofacial orthopedics. Am. J. Orthod..

[70] Wertz R.A. (1970). Skeletal and dental changes accompanying rapid midpalatal suture opening. Am. J. Orthod..

[71] Podesser B., Williams S., Crismani A.G., Bantleon H.P. (2007). Evaluation of the effects of rapid maxillary expansion in growing children using computer tomography scanning: A pilot study. Eur. J. Orthod..

[72] Garib D.G., Henriques J.F., Janson G., Freitas M.R., Coelho R.A. (2005). Rapid maxillary expansion—Tooth-tissue-borne vs. tooth-borne expanders: A CT evaluation of dentoskeletal effects. Angle Orthod..

[73] Kartalian A., Gohl E., Adamian M., Enciso R. (2010). Cone-beam computerized tomography evaluation of the maxillary dentoskeletal complex after rapid palatal expansion. Am. J. Orthod Dentofac. Orthop..

[74] Weissheimer A., Menezes L.M., Mezomo M., Dias D.M., Lima E.M., Rizzatto S.M. (2011). Immediate effects of rapid maxillary expansion with Haas-type and hyrax-type expanders: A randomized clinical trial. Am. J. Orthod. Dentofac. Orthop..

[75] Gunyuz Toklu M., Germec-Cakan D., Tozlu M. (2015). Periodontal, dentoalveolar, and skeletal effects of tooth-borne and tooth-bone-borne expansion appliances. Am. J. Orthod. Dentofac. Orthop..

[76] Akkaya S., Lorenzon S., Uçem T.T. (1998). Comparison of dental arch and arch perimeter changes between bonded rapid and slow maxillary expansion procedures. Eur. J. Orthod..

[77] Seubert B.J., Gaalaas L., Larson B.E., Grünheid T. (2021). Evaluation of transverse maxillary growth on cone-beam computed tomography images. Sci. Rep..

[78] Björk A., Skieller V. (1974). Growth in width of the maxilla studied by the implant method. Scand. J. Plast. Reconstr. Surg..

[79] Björk A., Skieller V. (1977). Growth of the maxilla in three dimensions as revealed radiographically by the implant method. Br. J. Orthod..

[80] Korn E., Baumrind S. (1990). Transverse development of the human jaws between the ages of 8.5 and 15.5 years, studied longitudinally with use of implants. J. Dent. Res..

[81] Hesby R.M., Marshall S.D., Dawson D.V., Southard K.A., Casko J.S., Franciscus R.G., Southard T.E. (2006). Transverse skeletal and dentoalveolar changes during growth. Am. J. Orthod. Dentofac. Orthop..

[82] Barone T.R., Cahali M.B., Vasconcelos C., Barone J.R. (2020). A comparison of tooth-borne and bone-anchored expansion devices in SARME. Oral. Maxillofac. Surg..

[83] Altug-Atac A.T., Karasu H.A., Aytac D. (2006). Surgically assisted rapid maxillary expansion compared with orthopedic rapid maxillary expansion. Angle Orthod..

